# A Review: The Bioactivities and Pharmacological Applications of *Phellinus linteus*

**DOI:** 10.3390/molecules24101888

**Published:** 2019-05-16

**Authors:** Wenhua Chen, Huiying Tan, Qian Liu, Xiaohua Zheng, Hua Zhang, Yuhong Liu, Lingchuan Xu

**Affiliations:** 1 School of Pharmacy, Shandong University of Traditional Chinese Medicine, Jinan 250355, China; cwh18363032223@163.com (W.C.); 18363036656@163.com (H.T.); cleanlq@163.com (Q.L.); 13465150376@163.com (X.Z.); 2 Key Laboratory of Medicinal Fungi and Resource Development in Shandong Province, Shandong University of Traditional Chinese Medicine, Jinan 250355, China

**Keywords:** *Phellinus linteus*, polysaccharides, polyphenols, biological activities, pharmacological applications

## Abstract

*Phellinus linteus* is a popular medicinal mushroom that is widely used in China, Korea, Japan, and other Asian countries. *P. linteus* comprises various bioactive components, such as polysaccharides, triterpenoids, phenylpropanoids, and furans, and has proven to be an effective therapeutic agent in traditional Chinese medicine for the treatment and the prevention of various diseases. A number of studies have reported that *P. linteus* possesses many biological activities useful for pharmacological applications, including anticancer, anti-inflammatory, immunomodulatory, antioxidative, and antifungal activities, as well as antidiabetic, hepatoprotective, and neuroprotective effects. This review article briefly presents the recent progress made in understanding the bioactive components, biological activities, pharmacological applications, safety, and prospects of *P. linteus*, and provides helpful references and promising directions for further studies of *P. linteus.*

## 1. Introduction

**Figure 1:**
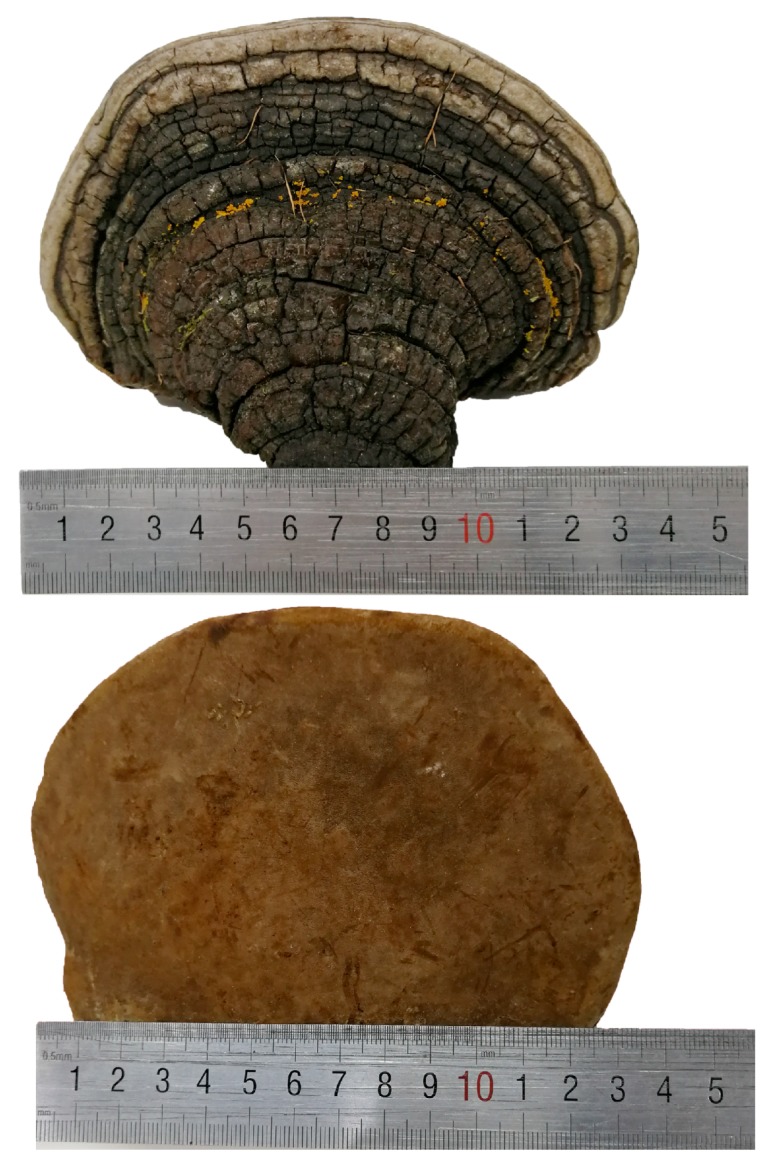
The fruiting body of *Phellinus linteus*.

*Phellinus linteus* (Berkeley & M. A. Curtis) Teng is a famous oriental medicinal mushroom, which belongs to *Phellinus* Quel., Hymenochaetaceae, Aphyllophorales, Hymenomycetes, and Basidiomycetes, and is more commonly known as “sanghuang” in China, “meshimakobu” in Japan, and “sangwhang” in Korea. Its basidiocarps are perennial, pileate, sessile, and usually horseshoe shaped. The pileal surface is dark brown when fresh and becomes black when dried, the pore surface is rusty brown when fresh and becomes brown when dried, the context is brown, and the upper context is a black carapace, and its tubes are cinnamon yellowish-brown when dried (Figure 1). *P. linteus* is a wood decay fungus that grows on the trunk of *Populus* Linn., *Quercus* Linn., *Toxicodendron vernicifluum* (Stokes) F. A. Barkley, and *Morus alba* Linn., and the best time for harvesting is from April to May. As a famous mushroom, *P. linteus* is mainly derived from tropical America, Africa, and East Asia, and it is particularly abundant in China, Japan, and Korea. Additionally, it has been recognized as beneficial to health and an ancient medicine for more than 2000 years [1]. *P. linteus* was first recorded in Shen Nong’s Herbal Classic (Shen Nong Ben Cao Jing), a famous Chinese medical book during the Han dynasty [2]. It has also appeared in many other Chinese medical books, including the New Compendium of Materia Medica (Xin Xiu Ben Cao) and Compendium of Materia Medica (Ben Cao Gang Mu) [3,4]. According to traditional Chinese medicine (TCM) theory, it was believed that *P. linteus* could be used to alleviate sickness in humans by consolidating a channel for hemostasis, removing blood-arthralgia consumption, relieving abdominal pain, and treating chronic diarrhea, among other benefits [5].

*P. linteus* plays a significant role in promoting health properties. This role has been attributed to the biological activity of its various components, including polysaccharides, triterpenoids, polyphenols, and pyrans. Based on modern pharmacological studies, *P. linteus* is reported to have multifaceted biological activities, including anti-inflammatory [6,7,8,9,10,11,12,13,14], immunomodulatory [15,16,17,18,19], antioxidative [20,21,22,23,24,25,26], antimicrobial, and antiviral [27,28,29,30,31,32,33], as well as anticancer [34,35,36,37,38,39,40,41,42,43,44,45,46,47,48,49,50,51,52,53,54,55,56,57], antidiabetic [58,59,60,61,62,63,64,65,66,67], hepatoprotective [68,69], and neuroprotective [70,71] effects. Among them, polysaccharides with β-glucan polymers are considered to be among the most important substances and are a potential candidate for developing novel anticancer drugs from natural products [72,73]. Meanwhile, polyphenols of *P. linteus* can also make a significant contribution in terms of their antitumor activity. All of the characterized polyphenols have demonstrated cytotoxic activities against various cancer cells, including pancreatic cancer stem cells, melanoma cells, NB4 human leukemia cells, human epithelial cancer line cells, human nasopharyngeal carcinomas cells, human nasopharyngeal carcinoma cells, hepatic stellate cells, HT29 human colon cancer cells, human breast cancer cells, human colon adenocarcinoma HCT116 cells, embryonic kidney carcinoma A293 cells, multiple myeloma U-266 cells, brain cancer cells, HepG2 cells, and human non-small cell lung carcinoma cells. On the basis of previous research [1,74,75,76,77], this paper presents a comprehensive and updated summary of the bioactive components, biological activities, pharmacological applications, possible molecular mechanisms, and safety of *P. linteus***.** In a word, it is also our hope that the current review will provide a helpful reference for further study and necessary information for improving the currently available therapeutic agents and dietary supplements of *P. linteus*.

## 2. Bioactive Components

**Figure 2:**
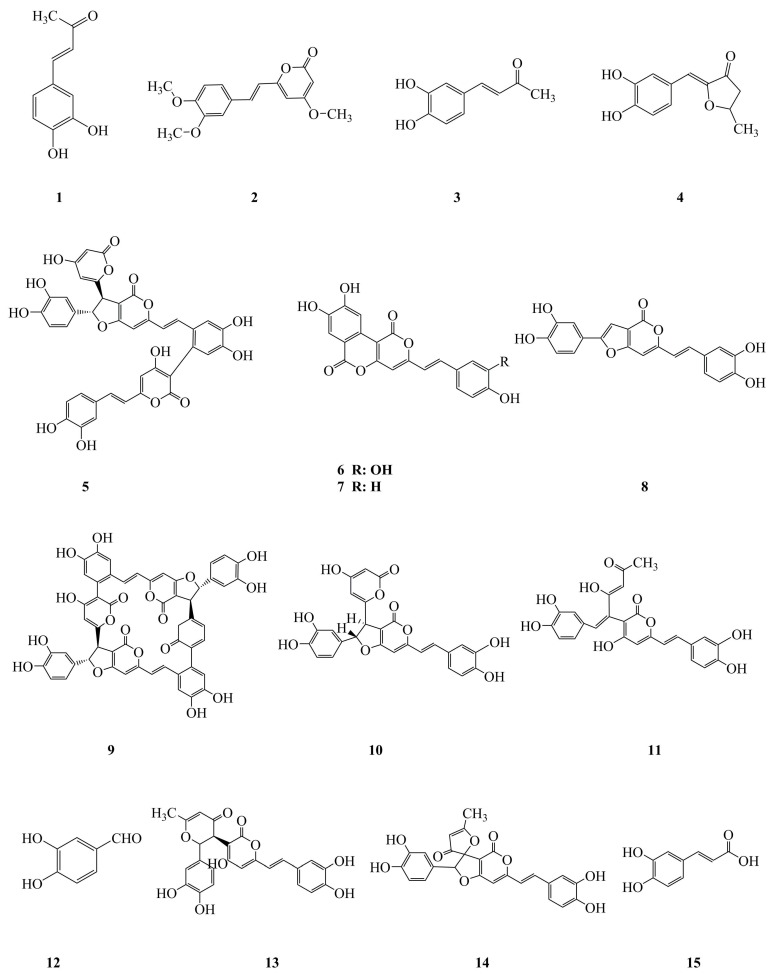

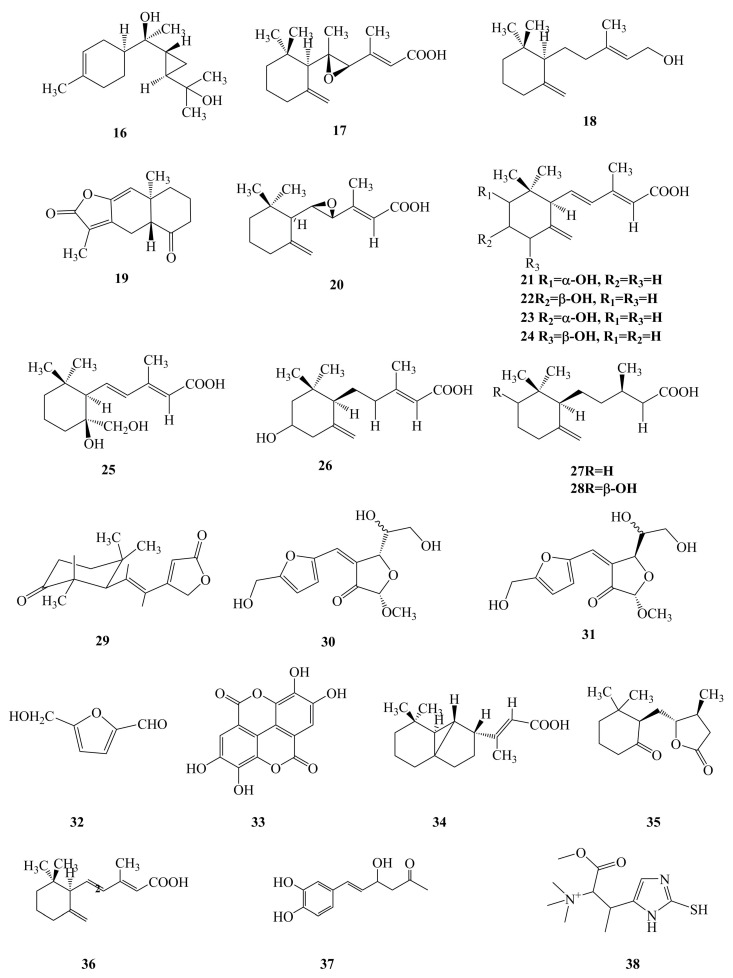
Chemical structures of bioactive components isolated from *P. linteus*. Phenylpropanoids (**1**–**15**), terpenoids (**16**–**28**), furans (**29**–**32**), and others (**33**–**38**).

It is obvious that the bioactive components of the fruiting body and the mycelium of *P. linteus* play a vital part in their biological activities and pharmacological applications. Phenylpropanoids (Figure 2, **1**–**15**), terpenoids (Figure 2, **16**–**28**), furans (Figure 2, **29**–**32**), and others (Figure 2, **33**–**38**) are believed to be the components responsible for the observed biological activities of *P. linteus*. The various groups of compounds have been summarized and are listed in Table 1. The specific chemical structures of these compounds are shown in Figure 2.

### 2.1. Phenylpropanoids

Phenylpropanoids are the most representative and predominant type of bioactive constituent in the fruiting body and the mycelium of *P. linteus* with verified anti-inflammatory [12], antioxidative [24], antitumor [53,55,56,57], antidiabetic [59,61,62,65,66,67], antimicrobial [28], antiviral [33], and anti-complementary activity [80], as well as cardioprotective [78] and gastroprotective [79] effects. Among them, 3,4-dihydroxybenzalacetone (**1**) from the fruiting body of *P. linteus* was reported to show anti-inflammatory activity [12]. It was reported that 3, 4-dihydroxybenzalacetone (**1**), hispidin (**2**), meshimakobnol A (**6**), meshimakobnol B (**7**), and phellifuropyranone A (**8**) showed antitumor effects in vitro and in vivo [53,55,56,57]. Some previous studies have indicated that hispidin (**2**), inotilone (**3**), 4-(3,4-dihydroxyphenyl)-3-buten-2-one (**4**), and caffeic acid (**15**) from the mycelium of *P. linteus* exhibited antioxidative activities [23,24]. Some articles have also shown that inotilone (**3**) and 4-(3,4-dihydroxyphenyl)-3-buten-2-one (**4**) have antiviral activities [33]. Recent studies have revealed that hispidin (**2**), phelligridimer A (**9**), hypholomine B (**10**), interfungin A (**11**), protocatechualdehyde (**12**), davallialactone (**13**), and inoscavin A (**14**) from the fruiting body of *P. linteus* all showed antidiabetic effects [59,61,62,65,66,67]. It was reported that phellinstatin (**5**) displayed antibacterial activity against *Staphylococcus aureus* and MRSA (Methicillin resistant *S. aureus*) [28]. In addition, hispidin (**2**) was reported to have a cardioprotective effect [78]. Furthermore, 4-(3,4-dihydroxyphenyl)-3-buten-2-one (**4**) was reported to exhibit gastric protective activity [79]. To date, 15 kinds of phenylpropanoid compounds have been isolated from *P. linteus* with biological activities and pharmacological applications. 

### 2.2. Terpenoids

Terpenoids are also the major bioactive constituents of the mycelium of *P. linteus* and are important secondary metabolites. To date, phytochemists have discovered 13 kinds of terpenoids from the mycelium of *P. linteus* with pharmacological activity [29,30,40,69]. It was reported that phellilane L (**16**), phellidene E (**17**), and (−)-*trans*-γ-monocyclofarnesol (**18**) exhibited antimicrobial activities against *P. gingivalis* [29,30]. In addition, atractylenolide I (**19**) was revealed to have antitumor activity [40]. Furthermore, phellinulin D (**20**), phellinulin E (**21**), phellinulin F (**22**), phellinulin G (**23**), phellinulin H (**24**), phellinulin I (**25**), phellinulin K (**26**), phellinulin M (**27**), and phellinulin N (**28**) isolated from the mycelium of *P. linteus* were indicated to have a hepatoprotective effect [69]. Furthermore, the chemical structure of terpenoid components is clearly correlated with pharmaceutical activities, and this relationship will be the subject of increasing research attention in the future.

### 2.3. Furans

Furans, a kind of pentacyclic compound containing oxygen, are an intermediate of synthetic drugs. Studies have demonstrated that phellinone (**29**) from the mycelium of *P. linteus* exhibited antimicrobial activities [31]. It has also been reported that phellinusfuran A (**30**) and phellinusfuran B (**31**) from the fruiting body of *P. linteus* showed anti-complementary activity [80]. Additionally, 5-hydroxymethyl-2-furaldehyde (**32**) from the fruiting body of *P. linteus* was shown to have antidiabetic effects [67].

### 2.4. Others

Ellagic acid (**33**) from the fruiting body of *P. linteus* was shown to have antidiabetic effects [65]. Phellilin C (**34**), γ-ionylideneacetic acid (**35**), and phellinulin A (**36**) from the mycelium of *P. linteus* exhibited a hepatoprotective effect [54,69]. Hispolon (**37**) from the mycelium of *P. linteus* exhibited anti-inflammatory activity [7,9]. Meanwhile, hispolon (**37**) from the fruiting body of *P. linteus* also showed antitumor activity [42]. In addition, ergothioneine (**38**) from the mycelium of *P. linteus* was reported to exhibit antidiabetic activity [62].

## 3. Biological Activities

### 3.1. Anti-Inflammatory Activities

Published reports have consistently indicated that some mushroom extracts exhibit anti-inflammatory effects. Furthermore, the bioactivities of medicinal and edible mushrooms have been shown to possess anti-inflammatory activity through suppression of the production of different types of inflammatory mediators [81,82]. Studies have shown that *n*-BuOH extracts from the fruiting body of *P. linteus* have anti-inflammatory activity through the inhibition of nitric oxide (NO), nitric oxide synthase (iNOS), and matrix metalloproteinase-9 (MMP-9) expression. These suppress the phosphorylation of protein kinase Cδ/NF-E2-related factor 2 (PKCδ/Nrf2)/antioxidant response element (ARE) signaling to upregulate the expression of heme-oxygenase-1 (HO-1) in lipopolysaccharide (LPS)-induced RAW264.7 macrophages in a time-dependent manner [6]. Several groups showed that hispolon (50, 100, 200, 400 µg/mL) from the mycelium of *P. linteus* possessed the greatest inhibitory effects against LPS-induced inflammatory mediators through suppressing the nuclear factor kappa beta (NF-κB) signaling pathway by reducing the expression of iNOS, NO, tumor necrosis factor (TNF)-α in RAW 264.7 cells, and murine primary peritoneal exudate macrophages (PEMs) [7]. Several in vitro and in vivo models stimulated by LPS and dextran sodium sulfate (DSS) from the mycelium of *P. linteus* extracts exhibited an anti-inflammatory effect through inhibiting the NF-κB and the mitogen-activated protein kinase (MAPK) signaling pathway by reducing the expression of iNOS, cyclooxygenase-2 (Cox-2), extracellular regulated protein kinase (ERK), and p38 [8]. Recent reports have indicated that hispolon of *P. linteus* inhibited LPS and lipoteichoic acid (LTA)-induced inducible iNOS/NO production through upregulating the HO-1 and downregulating the JNK/NF-κB signaling pathways in a time- and dose-dependent manner [9,10]. Studies have shown that ethyl acetate extracts (0–200 µg/mL) from the mycelium of *P. linteus* could inhibit β-hexosaminidase release and the activation of spleen tyrosine kinase (Syk), GRB2-associated-binding protein 2 (Gab2), and ERK expression in immunoglobulin E (IgE)/Ag-induced RBL-2H3 cells. It was also demonstrated that ethyl acetate extracts of *P. linteus* could inhibit LPS-induced NO production and pro-inflammatory cytokines in RAW 264.7 cells [11]. Moreover, 3,4-Dihydroxybenzalacetone (DBL) (5 mg/kg) from the fruiting body of *P. linteus* was reported to suppress the activation of MAPK and NF-κB by inhibiting the expression of Toll-like receptor 4 (TLR4) and phosphoinositide-3-kinase (PI3K)/AKT [12]. In addition, DBL not only markedly suppressed the protein expression of iNOS, COX-2, TNF-α, interleukin-1β (IL-1β), interleukin-6 (IL-6), MMP-2, and MMP-9, but also increased the expression of antioxidative enzymes such as superoxide dismutase (SOD), catalase, and glutathione peroxidase (GPx) [12]. The results indicated that DBL exhibited significant anti-inflammatory activity on LPS-induced acute lung injuries in mice. Many pharmacological studies have shown that polysaccharides (500 mg/kg/d) of *P. linteus* could markedly decrease the expression of inflammatory cytokines of IL-6, IL-1β, TNF-α, and iNOS in DSS-induced mice [13]. Another experiment indicated that polysaccharides (0–100 µg/mL) of *P. linteus* had potent anti-inflammatory activity through regulating the MAPK and the PPAR signaling pathways by reducing the level of TNF-α, IL-1𝛽, IL-6, NF-κB, activator protein-1 (AP-1), COX-2, iNOS, ERK1/2, p38, and JNK in LPS-induced RAW 264.7 macrophages [13]. Furthermore, it was reported that polysaccharides (0, 50, 100, 200 μmol/L) of *P. linteus* treated for 24, 48, and 72 h could downregulate the production of the pro-inflammatory cytokines of TNF-α, IL-1β, IL-2, IL-6, and IL-12, and upregulate the anti-inflammatory cytokines IL-4 and IL-10 through inhibiting the translocation of NF-κB in LPS-stimulated RAW264.7 macrophages [14]. All in all, the anti-inflammatory activities of *P. linteus* are closely related to the inhibition of pro-inflammatory mediators through the activation of the HO-1 signaling pathway and downregulation of the JNK–NF-κB–AP-1, reactive oxygen species (ROS), MAPK, and peroxisome proliferator-activated receptor (PPAR) signaling pathways. These anti-inflammatory effects suggest that *P. linteus* might hold promise for the treatment of inflammatory diseases. 

### 3.2. Immunomodulatory Activities

Some articles have shown that proteoglycan (0–500 μg/mL) from the fruiting body of *P. linteus* could enhance the expression of co-stimulatory molecules, CD80 and CD86, as well as the proliferation rates in B lymphocytes. The protein tyrosine kinase (PTK) and the protein kinase C (PKC) signaling pathways are also considered to be involved in the mechanism of these immunomodulatory activities [15]. A study confirmed that crude polysaccharides (200 mg/kg) from the fruiting body of *P. linteus* treated for four weeks exhibited immunostimulatory activity through enhancing the Th1-derived cytokine interferon-γ (IFN-γ) by T lymphocytes [16]. Another study demonstrated that a novel polysaccharide–protein complex extracted from *P. linteus* was an important biological response modifier of macrophages and NK cells and increased the proliferation of B cells in vitro [17]. It is well known that polysaccharides from *P. linteus* show strong immunomodulatory activity. One study showed that polysaccharides (100 μg/mL) from *P. linteus* treated for 6 and 24 h significantly decreased TNF-α, stimulated IL-10, and suppressed IL-6 [18]. A further study also reported that polysaccharides (50, 100, 200, 400 μg/mL) from *P. linteus* treated for 24 h could regulate the T helper 1 (Th1)/T helper 2 (Th2) balance through decreasing IFN-γ/IL-4 for the development of immunomodulatory efficacy [19]. However, the detailed mechanisms underlying this effect of *P. linteus* should be further explored.

### 3.3. Antioxidative Activities

The research conducted thus far has confirmed that extracts of *P. linteus* exhibit strong antioxidative activity in vitro and in vivo. Previous studies have found that polysaccharides (0.0625 mg/mL) of *P. linteus* treated for 2 h significantly attenuated tacrine-induced hepatotoxicity and mitochondria dysfunction by an antioxidant protective mechanism through reducing the production of ROS in HepG2 cells [20]. Wang et al. [21] evaluated the antioxidative activities of polysaccharides from the mycelium of *P. linteus* using the 1,1-diphenyl-2-picrylhydrazyl (DPPH) radical-scavenging activity, the Trolox-equivalent antioxidative capacity (TEAC), the ferric reducing ability of plasma (FRAP), and the cytoprotection tests in vitro. The antioxidative assays showed that the strong DPPH radical-scavenging capacities and the antioxidative activities occurred in a dose-dependent manner [21]. Some studies have revealed the antioxidative activity of the hispidin of *P. linteus* using DPPH, 2,2′-azino-bis(3-ethylbenzothiazoline-6-sulfonic acid) diammonium salt (ABTS), FRAP, hydroxyl radicals, and superoxide anion radicals assays. Therefore, hispidin of *P. linteus* could alleviate acrylamide-induced oxidative stresses through decreasing the ROS and the MMP and increasing glutathione (GSH) in Caco-2 cells, exhibiting potential antioxidative activity [22]. Another investigation reported that hispidin derivatives of *P. linteus* displayed notable free radical-scavenging activity. Theoretical results showed that the parameter of double bond dissociation enthalpy (BDE_d_) plays a central role in assessing the antioxidative activity values of hispidin derivatives [23]. Thermodynamic and kinetic investigations showed that the proton-coupled electron transfer (PCET) mechanism was associated with hispidin derivatives to scavenge free radicals [23]. Recently, some studies revealed that caffeic acid, inotilone, and 4-(3,4-dihydroxyphenyl)-3-buten-2-one (0.1–1 mM) from the mycelium of *P. linteus* showed dose-dependent antioxidative effects in ABTS and DPPH radical-scavenging activity assays, while phellilane H, (2E,4E)-(+)-4′-hydrox did not have any effects [24]. Yan et al. [25] revealed that the dose-dependent anti-aging activity of polysaccharides of *P. linteus* was closely related to the antioxidative activity in D-galactose-induced aging model mice. The results showed that polysaccharides (100, 200, and 400 mg/kg) of *P. linteus* treated for 40 days markedly increased the activity of SOD, catalase (CAT), and GSH-Px, and significantly reduced the malondialdehyde (MDA) content of serum and livers [25]. Administration of 300, 600, and 1200 mg/kg/d chitosan oligosaccharide (a natural polysaccharide) to aged rats induced using D-galactose (250 mg/kg/d) for eight weeks significantly increased the activity of CAT, GSH-Px, and SOD and decreased the content of MDA [26]. These results have revealed that the polysaccharides of *P. linteus* demonstrate marked anti-aging activity through increasing the antioxidant defenses and decreasing oxidative stress. Therefore, these findings indicate that *P. linteus* extracts may have potential as antioxidative agents.

### 3.4. Antimicrobial and Antiviral Activities

**Figure 3:**
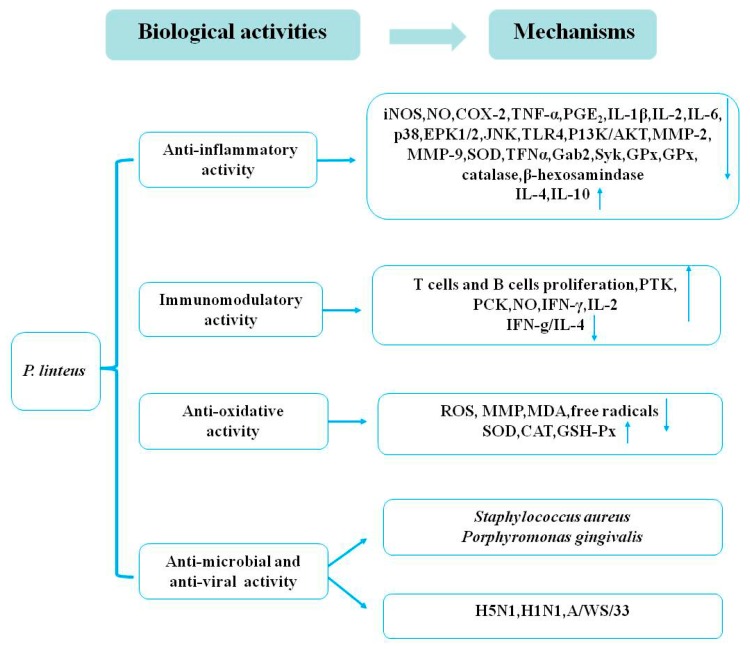
The biological activities of *P. linteus* and their mechanisms.

Hur et al. [27] revealed the antibacterial activity of different solvent extracts of *P. linteus* against 12 methicillin-resistant *S. aureus* stains. The results demonstrated that the *n*-BuOH fractions of *P. linteus* methanol extracts exhibited potent antimicrobial activity for all tested stains, with minimum inhibitory concentration (MIC) values ranging from 63 to 125 μg/mL [27]. In another study, a new trimeric hispidin derivative from the mycelium of *P. linteus*, phellinstatin, was reported to possess antibacterial properties [28]. The results suggested that phellinstatin significantly inhibited *S. aureus* enoyl-ACP reductase with an IC_50_ value of 6 μg/mL and exhibited potent antimicrobial activities against *S. aureus* and MRSA. An evaluation of the antimicrobial activity of phellilane L—isolated from the medicinal mushroom of *P. linteus*—against the periodontal bacteria *Porphyromonas gingivalis* had an MIC value of 278 μg/mL, lower than those of hinokitiol and triclosan that were used as positive controls (MIC of 25.0 and 3.13 μg/mL, respectively) in an antimicrobial assay [29]. γ-Ionylidene sesquiterpenoid from the mycelium of *P. linteus* was also shown to have antimicrobial activity against *P. gingivalis* [30]. The results revealed that (−)-*trans*-γ-monocyclofarnesol displayed significant antimicrobial activity against *P. gingivalis* with an MIC value of 5.9 μg/mL, while (+)-γ-ionylideneacetic acid and phellidene E had weak effects on the growth of *P. gingivalis*. Phellinone isolated from the mycelium of *P. linteus* was indicated to show antimicrobial activity against *Bacillus subtilis* IAM 1090 [31]. In addition, previous studies reported that extracts from the mycelium of *P. linteus* showed notable antiviral effects against influenza A virus H5N1 [32]. Inotilone and 4-(3,4-dihydroxyphenyl)-3-buten-2-one exhibited potent, dose-dependent, antiviral activity against H1N1 neuraminidase with IC_50_ values of 29.1 and 125.6 μg/mL, respectively [28]. They also exhibited an antiviral effect on influenza A/WS/33 virus with IC_50_ values of 61.5 and 52.3 μg/mL, respectively, with a positive control (oseltamivir IC_50_ = 64.7 μg/mL) in a viral cytopathic effect reduction assay in MDCK cells [33]. However, the active principle of these extracts and the detailed mechanism responsible for these antimicrobial and antiviral effects should be further investigated. Figure 3 shows all the known biological activities of *P. linteus* and their underlying mechanisms.

## 4. Pharmacological Applications

### 4.1. Anticancer Effects

**Figure 4:**
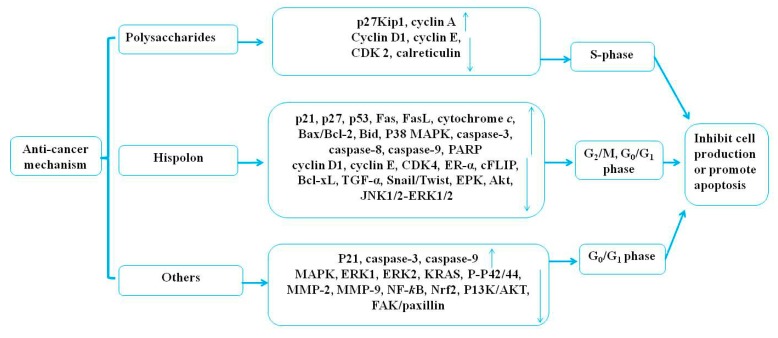
The potential anticancer mechanisms of *P. linteus*.

Medicinal mushrooms play an irreplaceable role regarding the therapeutic potential and the development of novel anticancer agents with no side effects. Joseph et al. (2017) revealed that the mechanisms of medicinal mushrooms involve the PI3K/AKT, the Wnt-CTNNB1, and the NF-κB signaling pathways in human cancers [83]. Sliva (2010) reviewed the alternative treatment of cancer using *P. linteus* [74]. In this review, the anticancer activities of *P. linteus* were summarized and are listed in Table 2 and Table 3. The potential anticancer mechanisms of *P. linteus* are presented in Figure 4.

#### 4.1.1. The Anticancer Effect of Polysaccharides

It has been known for centuries that the polysaccharides of medicinal mushrooms are the agents responsible for the observed anticancer properties. Wang et al. (2017) indicated that the bioactivity of the mushroom polysaccharides exhibits a close relationship with the structure of monosaccharide composition and the biosynthesis pathway [84]. A series of studies have shown that mushroom polysaccharides with β-glucans, β-(1→3) linkages, and water solubility exhibit more anticancer ability [72,73]. The proposed mechanism by which mushroom polysaccharides exert their antitumor effect includes cancer-preventing activity, immuno-enhancing activity, and direct tumor-suppressing activity [85].

The polysaccharides of *P. linteus* have shown anticancer activities both in vitro and in vivo. One study found that polysaccharides (100, 200 mg/kg/d) of *P. linteus* treated for 30 days significantly inhibited tumor growth in a human colorectal carcinoma cell (HT29)-bearing mouse model in vivo compared with saline (0.2 mL/d) as a negative control and cisplatin (2 mg/kg/d) as a positive control [34]. Compared with the untreated control, polysaccharides (25, 50, 100, 200 μg/mL) from the fresh fruiting body of *P. linteus* treated for 1.5 to 48 h significantly inhibited the proliferation of HT29 by blocking the cell cycle in S-phase and downregulating the expression of cyclin D1, cyclin E, and cyclin-dependent kinases (CDK2) with increased upregulation of P27kip1 expression in a dose-dependent manner but with no contribution to apoptosis and autophagy [34]. Some other studies have also shown that polysaccharides (50, 100, 200 μg/mL) from the fresh fruiting body of *P. linteus* treated for 24, 48, and 72 h caused significant inhibition of HepG2 cell proliferation in a dose-dependent manner (48 h, IC_50_ = 125 μg/mL). Furthermore, administration of 100 and 200 mg/kg of *P. linteus* polysaccharide for 18 days showed a significant inhibitory effect on tumor growth in vivo compared with the tumor-bearing (TB) control mice. The results have generally indicated that polysaccharides of *P. linteus* exhibit significant antitumor activity against HepG2 through blocking tumor cells going into the S stage, upregulating the expression of P27kip1 and cyclin A, and downregulating the expression of calreticulin, cyclin D1, cyclin E, and CDK2 in vitro and in vivo, but there is an urgent need to verify whether there is a relationship between this remarkable decrease in CDK2 and induced S-phase arrest [35]. Pei et al. (2013) revealed a novel high molecular weight polysaccharide (PL-N1) (50, 100, 200 μg/mL) from the mycelium of *P. linteus* composed of arabinose, xylose, glucose, and galactose, and the backbone of PL-N1 was (1→4)-linked β-D-xylopyranosyl residues; it could inhibit the growth of HepG2 cells in a dose-dependent manner in vitro [36]. Mei et al. (2015) found two novel high molecular weight polysaccharides—PLPS-1 with a backbone of α-D-α-Glc (1→4)-α-D-Glc (1→6) units and PLPS-2 with the backbone of α-(1→3)-D-Glc and α-(1→6)-D-Glc. The study indicated that PLPS-1 (25, 50, 100, 200 μg/mL), but not PLPS-2, from the mycelium of *P. linteus* exhibited strong anticancer activity against S-180 sarcoma cells by induction of apoptosis, where 5-fluorouracil (5-Fu) (25 μg/mL) was used as a positive control [37]. Protein–protein interaction (PPI) analysis has indicated that the DJ-1 and the 14-3-3 proteins play important roles in polysaccharide-treated HepG2 cells with β-actin as a positive control, but whether these proteins can be used for markers of hepatocellular carcinoma (HCC) needs further investigation [38]. The present study showed that the polysaccharides (50 μg/mL) of *P. linteus* could reduce the side effects of camptothecin 11 (CPT 11) (10 ng/ml) when they were used as a drug combination in colon cancer HCT116 and HT29 cells [39]. It was reported that ethanol extracts, *n*-hexane fractions, and ethyl acetate fractions of *P. linteus* mycelia inhibited the growth of the HT29 cell line in a dose-dependent manner, and the IC_50_ values were 149.9, 69.8, and 77.8 µg/mL, respectively [40]. According to these studies, polysaccharides of *P. linteus* played an important role in producing the observed anticancer activities.

#### 4.1.2. The Anticancer Effect of Hispolon

Hispolon, a phenol compound isolated from the fruiting body of *P. linteus*, plays an important role in facilitating anticancer activity. Recent studies have shown that the apoptotic mechanism of hispolon is to inhibit cell proliferation and promote cell apoptosis through blocking G/G1 to S transition in NB4 human leukemia cells in a time- and dose-dependent manner [41]. The results have indicated that hispolon (10 μg/mL, 24 h) of *P. linteus* induced cell arrest through increasing the protein levels of p53, p21, and p27 and reducing the protein levels of cyclin D1, cyclin E, CDK 2, and CDK 4. Furthermore, it could increase the expressions of the extrinsic apoptotic proteins of Fas and FasL, intrinsic proteins of cytochrome *c*, and the ratio of Bax/ Bcl-2 [41]. On the one hand, studies have revealed that hispolon of *P. linteus* could decrease the content of melanin in α-melanocyte-stimulating hormone (α-MSH) stimulated through suppressing tyrosinase and the microphthalmia-associated transcription factor (MITF) expression with a concentration of less than 2 μg/mL in B16-F10 cells. On the other hand, it could also induce cell apoptosis by increasing the expression of caspase-3, -8, and -9, at concentrations greater than 10 μg/mL in B16-F10 cells, but not in Detroit 551 normal fibroblast cells [42]. In the present study, the studies showed that hispolon (0–100 μM) of *P. linteus* could inhibit cell proliferation and directly induce cell apoptosis by promoting the phosphorylation of JNK1/2, ERK1/2, and p38 MAPK to activate Csp-3, Csp-8, Csp-9, and Poly ADP-Ribose Polymerase (PARP) expression in a dose- and time-dependent manner when used on HONE-1 and NPC-039 human nasopharyngeal carcinoma cells [43]. Previous studies have suggested that hispolon (0–100 μM; IC_50_ = 70 μM) of *P. linteus* induced cell apoptosis through increasing PARP cleavage and decreasing the expression of Bcl-2. Moreover, it could also inhibit cell proliferation by reducing ER-α expression at the level of both mRNA and protein in MCF7 and T47D human breast cancer cells in vitro, but this needs to be further studied in vivo [44]. Previous reports suggested that hispolon (70 μM) of *P. linteus* could promote tumor necrosis factor-related apoptosis-inducing ligand (TRAIL)-induced cell apoptosis through downregulation of the survival proteins of cFLIP, Bcl-2, and Bcl-xL, with the reactive oxygen species–extracellular signal-regulated–kinase-homologous protein (ROS–ERK–CHOP) pathway and upregulation of the expression of Bax and truncated Bid, as well as the death receptors of p53, in a dose- and time-dependent manner. Meanwhile, it could also be sensitive to the expression of caspase 3, caspase 8, and caspase 9 on human colon cancer cells of HCT116 in vitro [45]. Current studies provide strong evidence that hispolon (1–500 mM; IC_50_ = 65 μM) of *P. linteus* inhibits cell proliferation through repressing the transforming growth factor β (TGF-β)-Snail/Twist signaling pathway of epithelial–mesenchymal transition (EMT) in human epithelial cancer cells in vitro compared with the untreated control group [46]. Modern pharmacological research has shown that hispolon (25, 50 μM) of *P. linteus* used for 24, 48, and 72 h significantly inhibited tumor cell proliferation and promoted cell apoptosis in glioblastoma U87MG cells. Among them, p53 plays a vital role in inducing cell cycle arrest as well as blocking G2/M-phase and reducing the expression of cyclin D4 while increasing the expression of CDK inhibitor p21 compared with a control group [47].

All in all, hispolon of *P. linteus* has been shown to exhibit therapeutic efficacy against various cancer cells, including colorectal, melanoma, leukemia, nasopharyngeal, breast, epithelial, and glioblastoma cancer cells. The mechanism behind the anticancer activities of hispolon of *P. linteus* anticancer activities works through blocking the cell cycle in G_0_/G_1_-phase or G_2_/M-phase through inhibiting cell proliferation and inducing cell apoptosis. Therefore, hispolon is an effective potential anticancer agent, but researchers have only focused on the induction of antiproliferative effects and apoptosis in tumor cells in vitro. Therefore, there is an urgent need to further verify the results by carrying out experiments in vivo.

#### 4.1.3. The Anticancer Effect of Others

Medicinal mushrooms, as natural products, have been applied to treat cancer for millennia. Some experts revealed the anticancer activities of ethanol extracts (300 mg/kg/d) from the mycelium of *P. linteus* in combination with *Inonotus obliquus*, *Antrodia camphorata*, and *Ganoderma lucidum* for the Cell Activation Research Institute (CARI III), reducing tumor weight compared with the doxorubicin (Dox)-treated group and increasing life span (ILS% = 50.88%) compared with the tumor control group. The results also indicated that they displayed antiproliferative activity against B16F10 melanoma cells through inducing G0/G1 cell cycle arrest by decreasing cyclin D1 and CDK2 expression and inducing p21 in vitro and in vivo [48]. The present study showed that extracts (0–700 µg/mL) of *P. linteus* could induce apoptosis through oxidative stress by stimulating Csp-3 and Csp-9 in a variety of human malignancies, including prostate cancer metastasized to bone (PC-3), prostate cancer metastasized to brain (DU-145), prostate cancer metastasized to lymph nodes (LNCaP), bladder cancer T24, kidney cancer ACHN, lung cancer A549, breast cancer MCF-7, stomach cancer AGS, liver cancer HepG2, and brain cancer U-87 cells, compared with an untreated control [49]. However, the results did not specify which extracts were used, and further research has been limited. Previous studies revealed that aqueous extracts from the fruiting body of *P. linteus* exhibited a dose-dependent antiproliferative effect, with an IC_50_ value of 40 mg/mL in MDA-MB-231 breast cancer cell lines [control: 10 µg/mL 5-flurouracil (5-FU) in vitro] [50]. Ethanolic extracts (100 µg/mL) of *P. linteus* and cetuximab (10, 30 µg/mL) that were combined for three days of treatment could work against G12VKRAS mutant colon cancer cells by inducing apoptosis. Therefore, the results have concluded that ethanolic extracts of *P. linteus* significantly improved cetuximab resistance in Kirsten rat sarcoma viral oncogene homolog (KRAS)-mutant colon cancer through modulating the RAS/MAPK pathways by reducing the phosphorylation of MAPK, ERK1, and ERK2 by decreasing the expression of KRAS and p-p42/44 MAPK to promote apoptosis. Further pharmacological experiments have also shown that ethanol extracts from the mycelium of *P. linteus* (400 mg/kg/d) treated for 23 days inhibited proliferation with a tumor-xenografted mouse model compared with a cetuximab (10, 30 mg/kg/d) control group [51]. Moreover, current evidence has indicated that extracts of *P. linteus* administered orally at a dose of 1100 mg three times per day could assist in the chemotherapeutic treatment of pancreatic ductal adenocarcinoma and improve patient survival according to a clinical study of 323 patients from January 1995 to December 2014. However, well-designed randomized control trials are needed to evaluate and confirm this hypothesis [52]. Previous studies have demonstrated that 3,4-dihydroxybenzalactone (0, 6.25, 12.5, 25, 50 µM) from the fruiting body of *P. linteus*, treated for 24 h inhibited the migratory and the invasive abilities of cancer cells through suppressing the enzymatic activity of MMP-2 and MMP-9, decreasing the activity of PI3K/AKT, MAPKs, and focal adhesion kinase (FAK)/paxillin, influencing EMT/Snail and Slug, and affecting the NF-κB and Nrf2 signaling pathways in human non-small cell lung carcinoma A 549 cells [53]. Recent studies have found that phellinulin A (40 µM) from the mycelium of *P. linteus* had significant inhibitory and therapeutic effects on hepatic fibrosis, especially in activated rat hepatic stellate cells [54]. Numerous recent reports have shown that atractylenolide I (0–100 µg/mL) from the mycelium of *P. linteus* had good preventive and therapeutic effects on HT29 human colon cancer cells and acted in a dose-dependent manner [40]. Recent studies have suggested that hispidin (50, 100, 150 μM) from the mycelium of *P. linteus* treated for 24, 48, and 72 h had therapeutic potential against BxPC-3 pancreatic cancer cells and cancer stem cells (CSCs) by downregulating the expression of NF-ĸB in a dose-dependent manner in vitro, and that it exerted a synergistic effect with gemcitabine [55]. Furthermore, phellifuropyranone, meshimakobnol A, and meshimakobnol B isolated from the fruiting body of *P. linteus* exhibited antiproliferative activity against mouse melanoma cells and human lung cancer cells in vitro, with 50% growth-inhibitory concentrations (GI_50_) of 5.6–31.3, 7.1–22.6, and 6.1–15.0 μM, respectively, compared with paclitaxel as a positive control [56,57]. However, the antitumor mechanism underlying this needs to be further examined in animal models.

### 4.2. Hypoglycemic Effects

**Figure 5:**
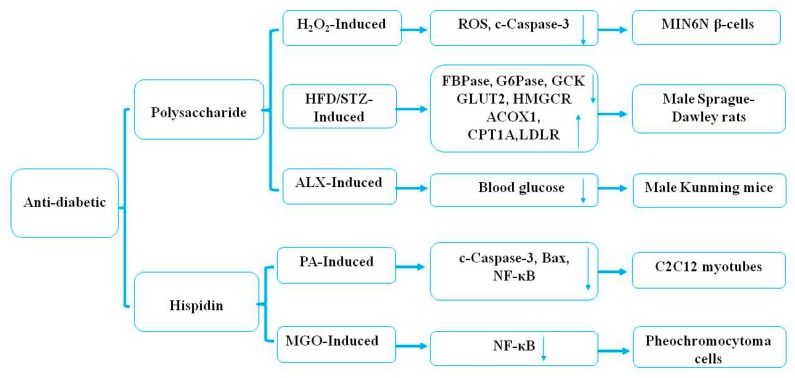
The potential mechanism of the antidiabetic effect of *P. linteus.*

Some previous studies have indicated that natural health products have potential antidiabetic effects [86]. Kim et al. [58] demonstrated that polysaccharides (30 mg/kg) of *P. linteus*, as examined every other day from eight to 24 weeks, markedly increased Th2 cytokine production and decreased Th1 cytokine production in a Streptozotocin-induced diabetic animal model. In addition, polysaccharides from the mycelium of *P. linteus* inhibited autoimmune diabetes through reducing the expression of cytokines, including IFN-γ, IL-2, and TNF-α in Th1 cells and macrophages in vitro [58]. One study confirmed that treatment with hispidin (70 μM) from the mycelium of *P. linteus* could not only exhibit potent-free radical-scavenging effects but could also protect MIN6N β-cells from ROS toxicity through inhibiting cell apoptosis and caspase-3 activity induced by hydrogen peroxide in diabetes [59]. Oral administration with polysaccharides from the mycelium of *P. linteus* at 100 mg/kg body weight/d could markedly decrease the level of blood glucose by 35.60% in alloxan (ALX)-induced diabetic mice [60]. The mechanism of the hypoglycemic effect may be related to the chemical structure of the polysaccharides and needs further study. Another study showed that hispidin (1–100 μM) from the mycelium of *P. linteus* treated for 8 h could exhibit a cytoprotective effect through reducing the expression of Bax, ROS, and NF-κB in palmitate (PA)-induced oxidative stress in C2C12 myotubes, and that it had therapeutic potential as an antidiabetic drug [61]. In vitro studies have indicated that ergothioneine (2 μM) and hispidin (2 μM) from the mycelium of *P. linteus* had hypoglycemic effects by inhibiting the expression of the NF-κB signaling pathway through antioxidative activities in rat pheochromocytoma cells compared with positive controls [epalrestat (EPA), 10 μM; aminoguanidine (AMG), 100 nM] [62]. Recent studies have demonstrated the underlying molecular mechanisms of polysaccharides of *P. linteus* in the treatment of diabetes mellitus [63]. Polysaccharides (50 mg/kg) of *P. linteus* examined twice a day for four weeks significantly decreased blood glucose and improved glucose intolerance in male C57BL/6J mice compared with blank and high-fat high-fructose diet (HFD) control groups. In addition, polysaccharides of *P. linteus* ameliorated insulin resistance through regulating hepatic phospholipid metabolism and stimulating insulin signaling transduction [63]. It was reported that polysaccharides (300, 600 mg/kg) from the mycelium of *P. linteus* treated for eight weeks efficiently decreased the blood glucose level and increased insulin resistance and glycogen in an HFD-induced (120 mg/kg) storage and low-dose streptozotocin (STZ) (45 mg/kg)-induced type 2 diabetic rat model [64]. The above research results have shown that polysaccharides from the fruiting body of *P. linteus* have a better application potential in the adjuvant therapy of type 2 diabetes. It was reported that phelligridimer A, protocatechualdehyde, davallialactone, hypholomine B, interfungin A, and inoscavin A isolated from the fruiting body of *P. linteus* possessed significant rat lens aldose reductase and human recombinant aldose reductase inhibitory activity in vitro, with IC_50_ values of 0.63, 4.26, 20.52, 0.33, 0.82, 1.03, and 1.06 μM and 1.37, 7.93, 35.36, 0.56, 1.28, 1.82, and 1.40 μM, respectively [65]. Protocatechualdehyde, davallialactone, and inoscavin A isolated from the fruiting body of *P. linteus* showed a significant inhibitory effect on methylglyoxal-medicated protein modification with IC_50_ values of 144.28, 213.15, and 158.66 μM, respectively [66]. Protocatechualdehyde and 5-hydroxymethyl-2-furaldehyde isolated from the fruiting body of *P. linteus* had an inhibitory effect on the oxidation of catalytic L-tyrosine with an IC_50_ value of 0.40 and 90.8 mg/mL, respectively [67]. These findings demonstrated that protocatechualdehyde, davallialactone, hypholomine B, interfungin A, inoscavin A, and 5-hydroxymethyl-2-furaldehyde all had a potential therapeutic effect on diabetes [65,66,67]. The potential mechanism underlying the antidiabetic effects of *P. linteus* is presented in Figure 5.

### 4.3. Hepatoprotective Effects

Nowadays, polysaccharides from plants and fungi are attracting more and more attention due to their hepatoprotective activities [87]. Previous studies have revealed that a polysaccharide from the wild fruiting body of *P. linteus* at a dose of 50 mg/kg body weight twice a day could protect against thioacetamide (TAA)-induced liver fibrosis through the regulation of oxidative stress pathways by increasing the expression of cysteine, GSH, GSTA4, hemoglobin-heme, and by decreasing the expression of hemopexin, free-heme, free-iron, methionine, heat shock pathways, and metabolic pathways for amino acids and nucleic acids in rats [68]. The studies presented here demonstrated that ethanol extracts of *P. linteus* at a dose of 20 mg/kg exhibited a protective effect against liver injury induced by dimethyl nitrosamine (DMN) in hepatic fibrosis Wistar rats. Furthermore, phellinulin A, phellinulin D, phellinulin E, phellinulin F, phellinulin G, phellinulins H, phellinulin I, phellinulin K, phellinulin M, phellinulin N, phellilin C, and γ-ionylideneacetic acid from the mycelium of *P. linteus* all showed inhibitory activities at a concentration of 40 µM against activated rat hepatic stellate cells (HSCs), with inhibition rates of 67.2%, 4.2%, 23.6%, 26.7%, 15.2%, 60.6%, 50.9%, 67.9%, 56.4%, 47.2%, 24.2%, and 39.7%, respectively, compared with silymarin (36.4%) as a positive control [69]. Above all, *P. linteus* has traditionally been used as a dietary supplement or traditional Chinese medicine to treat hepatitis, and the above studies might provide a scientific explanation underlying the traditional application.

### 4.4. Neuroprotective Effects 

Recently, researchers have carried out some investigations on *P. linteus* extracts used in the central nervous system. Polysaccharides from the fruiting body of *P. linteus* have played an important role in age-related neurodegenerative diseases [70]. These findings suggested that polysaccharides (200, 375, 750 μg/mL) of *P. linteus* treated for 24 h induced human leukemia cell apoptosis by increasing the expression of ROS and MMP in THP-1 monocytes in a dose-dependent manner [70]. Later, another interesting study reported that inotilone and 4-(3,4-dihydroxyphenyl)-3-buten-2-one of *P. linteus* exhibited potent neuraminidase inhibitory activity [71]. These preliminary analyses have shown that ethyl acetate extracts (0.1–5 μg/mL) of *P. linteus* had dose-dependent neuroprotective effects on oxidative stress (H_2_O_2_)-induced apoptosis through inhibiting the cleavage of caspase-3, PARP, and MAPKs by suppressing the level of ROS and increasing the expression of HO-1, CAT, GPx-1, and SOD in SK-N-MC cells [71]. However, *P. linteus* extracts should be investigated in an in vivo model in order to test their potential to prevent or ameliorate neurodegenerative diseases.

### 4.5. Others

Water extracts (1.725 mL/kg/d) from the fruiting body of *P. linteus* were reported to exhibit a significant therapeutic effect on prostatitis after 30 days through inhibiting benign prostatic hyperplasia by suppressing the dihydrotestosterone activity of testosterone involved in the conversion by enzyme 5-reductase in vivo compared with finasteride (1 mL/kg/d) as a positive control [88]. Previous investigations indicated that hispidin (3, 10, 30 μM) of *P. linteus* could protect cardiomyoblast cells against H_2_O_2_-induced apoptosis through suppressing the Akt/GSK-3β and ERK1/2 signaling pathway by inhibiting the expression of caspase-3 and Bax and increasing the expression of Bcl-2 in H9c2 cardiomyoblast cells in a dose-dependent manner [78]. Polysaccharides of *P. linteus* at doses of 0, 5, 10, and 20 μg/mL could prevent atherosclerosis through increasing cholesterol efflux by the PPAR-γ/adenosine 5′-triphosphate (ATP)-binding cassette transporter A1 (ABCA1)/ATP-binding cassette G1 (ABCG1) signaling pathway in oxidized low-density lipoprotein-loaded THP-1 macrophages, while polysaccharides (100 μg/mL) of *P. linteus* reduced cholesterol efflux by inducing mitochondrial dysfunction and oxidative stress [89]. Furthermore, the studies have clearly revealed that 4-(3,4-dihydroxyphenyl)-3-buten-2-one (1, 5, 10 µg/kg) effectively prevented naproxen-induced gastric antral ulcers in rats by the prevention of lipid peroxidation and the activation of radical-scavenging enzymes in a dose-dependent manner [79]. The 28-day oral administration of extracts (3%) from the mycelium of *P. linteus* against ultraviolet B- induced hyperpigmentation due to the reduced melanin pigment content and PI3K/Akt/glycogen synthase kinase-3beta (GSK3 β) phosphorylation, indicating that the *P. linteus* extracts possessed anti-pigmentation effects in B16F0 melanoma cells compared with arbutin (2%) as a positive control [90]. Furthermore, two novel stereoisomers of furan derivatives, phellinusfurans A and B, which were isolated from the fruiting body of *P. linteus*, showed a strong anti-complementary effect with IC_50_ values of 33.6 and 33.7 μM, respectively, compared with rosmarinic acid (IC_50_ 180 μM) that was used as a positive control [80]. In brief, *P. linteus* has shown various pharmacological benefits and can be used as an alternative or an adjuvant treatment for a variety of diseases.

## 5. Safety

Previous studies have indicated that polysaccharides of *P. linteus* administered at a dose of 200 mg/kg for 30 days did not significantly enhance the level of ALT and AST in serum, LPO in the liver and kidney, nor did it display toxicity [34]. An acute toxicity study has demonstrated that the mycelium of *P. linteus*, in combination with *Inonotus obliquus*, *Antrodia camphorata*, and *Ganoderma lucidum* ethanol extracts, given orally at a dose of 1000 mg/kg/d for 14 days did not exhibit any mortality or signs of toxicity compared with the vehicle control group [48]. However, it has not been reported which major monomer components from the mycelium of *P. linteus* produce bioactive activities and synergistic effects with the mycelium of three other fungi. Studies have evaluated ethanol extracts of *P. linteus* administration at a dose of 400 mg/kg for 23 days, which significantly exhibited a synergistic anticancer effect and had no adverse effect [51]. Compared to the levels in whole *P. linteus*, β-glucan (29.20 mg/g) showed a synergistic anticancer effect by overcoming cetuximab resistance. Thus, the results concluded that the administration of extracts from the mycelium of *P. linteus* for 23 days did not cause obvious complications. However, going through the available literature, it can be concluded that there is a lack of chronic toxicity studies, and there are still research gaps with regard to its mode of action and its pharmaceutical standardization.

## 6. Conclusions and Future Perspectives

*P. linteus* is one of the most important and frequently used traditional fungal medicines. It has been traditionally used to consolidate channels for hemostasis, remove blood-arthralgia consumption, relieve abdominal pain, and treat chronic diarrhea in China. Recently, *P. linteus* has attracted increasing scientific interest. Furthermore, a variety of functional supplements made with *P. linteus* have been developed in China, Japan, and Korea. However, certain aspects still need to be further studied and explored.

It is common knowledge that the bioactive compounds in both fruiting bodies and mycelial extracts of *P. linteus* produce beneficial responses regarding human health. Moreover, several studies have been published where *P. linteus* shows multiple functions against various diseases, especially against various types of cancer. The results indicated that polysaccharides and hispolon from *P. linteus* could become potential candidates for the treatment of various cancers in the future. However, there is no uniformity or predictability in terms of the bioactive compounds and the mechanism of biological activities of *P. linteus*. Therefore, further studies should focus on the relationship between the structure and the antitumor activity, clarify their anticancer mechanisms at the molecular level, and improve the different biological activities by means of the chemical modification of *P. linteus* for its safe application in human healthcare [9]. In addition, the relationship between the structural characteristics of homogeneous polysaccharides and the bioactivity of *P. linteus* also needs to be explored and illustrated. Furthermore, the biological characteristics of *P. linteus* are well understood, but most of the current studies are limited to the crude extract and a small amount of pure isolated compounds. These findings have concluded that many bioactive compounds are yet to be identified. Therefore, much research needs to be conducted in plant identification, extraction, isolation, and identification of other bioactive compounds to fully confirm its traditional uses and provide evidence to rationalize its use in humans. Finally, modern pharmacological studies have indicated that *P. linteus* has a number of biological and pharmacological activities, including anti-inflammatory, immunomodulatory, antioxidative, antimicrobial, and antiviral activities, as well as anticancer, hypoglycemic, hepatoprotective, and neuroprotective effects. Therefore, there is an urgent need to evaluate its safety and efficacy to ensure its clinical application.

## Figures and Tables

**Table 1:** The bioactive components isolated from *P. linteus*.

No.	Compound Name	Classification	Origin	Biological Activity	References
1	3,4-Dihydroxybenzalacetone	Phenylpropanoid	Fruiting body of *P. linteus*	Anti-inflammatory, antitumor	[12,53]
2	Hispidin	Phenylpropanoid	Mycelium of *P. linteus*	Antioxidative, antitumor, antidiabetic, cardioprotective	[22,23,55,59,61,62,78]
3	Inotilone	Phenylpropanoid	Mycelium of *P. linteus*	Antioxidative, antiviral	[24,33]
4	4-(3,4-Dihydroxyphenyl)-3-buten-2-one	Phenylpropanoid	Mycelium of *P. linteus*	Antioxidative, antiviral, gastroprotective	[24,33,79]
5	Phellinstatin	Phenylpropanoid	Mycelium of *P. linteus*	Antimicrobial	[28]
6	Meshimakobnol A	Phenylpropanoid	Fruiting body of *P. linteus*	Antitumor	[56,57]
7	Meshimakobnol B	Phenylpropanoid	Fruiting body of *P. linteus*	Antitumor	[56,57]
8	Phellifuropyranone A	Phenylpropanoid	Fruiting body of *P. linteus*	Antitumor	[57]
9	Phelligridimer A	Phenylpropanoid	Fruiting body of *P. linteus*	Antidiabetic	[65]
10	Hypholomine B	Phenylpropanoid	Fruiting body of *P. linteus*	Antidiabetic	[65]
11	Interfungin A	Phenylpropanoid	Fruiting body of *P. linteus*	Antidiabetic	[65]
12	Protocatechualdehyde	Phenylpropanoid	Fruiting body of *P. linteus*	Antidiabetic	[65,66]
13	Davallialactone	Phenylpropanoid	Fruiting body of *P. linteus*	Antidiabetic	[65,66]
14	Inoscavin A	Phenylpropanoid	Fruiting body of *P. linteus*	Antidiabetic	[65,66]
15	Caffeic acid	Phenylpropanoid	Mycelium of *P. linteus*	Antioxidative	[24]
16	Phellilane L	Terpenoid	Mycelium of *P. linteus*	Antimicrobial	[29]
17	Phellidene E	Terpenoid	Mycelium of *P. linteus*	Antimicrobial	[30]
18	(−)-*trans*-γ-Monocyclofarnesol	Terpenoid	Mycelium of *P. linteus*	Antimicrobial	[30]
19	Atractylenolide I	Terpenoid	Mycelium of *P. linteus*	Antitumor	[40]
20	Phellinulin D	Terpenoid	Mycelium of *P. linteus*	Hepatoprotective	[69]
21	Phellinulin E	Terpenoid	Mycelium of *P. linteus*	Hepatoprotective	[69]
22	Phellinulin F	Terpenoid	Mycelium of *P. linteus*	Hepatoprotective	[69]
23	Phellinulin G	Terpenoid	Mycelium of *P. linteus*	Hepatoprotective	[69]
24	Phellinulin H	Terpenoid	Mycelium of *P. linteus*	Hepatoprotective	[69]
25	Phellinulin I	Terpenoid	Mycelium of *P. linteus*	Hepatoprotective	[69]
26	Phellinulin K	Terpenoid	Mycelium of *P. linteus*	Hepatoprotective	[69]
27	Phellinulin M	Terpenoid	Mycelium of *P. linteus*	Hepatoprotective	[69]
28	Phellinulin N	Terpenoid	Mycelium of *P. linteus*	Hepatoprotective	[69]
29	Phellinone	Furan	Mycelium of *P. linteus*	Antimicrobial	[31]
30	Phellinusfuran A	Furan	Fruiting body of *P. linteus*	Anti-complementary	[80]
31	Phellinusfuran B	Furan	Fruiting body of *P. linteus*	Anti-complementary	[80]
32	5-Hydroxymethyl-2-furaldehyde	Furan	Fruiting body of *P. linteus*	Antidiabetic	[67]
33	Ellagic acid	Other	Fruiting body of *P. linteus*	Antidiabetic	[65]
34	Phellilin C	Other	Mycelium of *P. linteus*	Hepatoprotective	[69]
35	γ-Ionylideneacetic acid	Other	Mycelium of *P. linteus*	Hepatoprotective	[69]
36	Phellinulin A	Other	Mycelium of *P. linteus*	Hepatoprotective	[54,69]
37	Hispolon	Other	Fruiting body and mycelium of *P. linteus*	Antitumor, anti-inflammatory	[7,9,41,42,43]
38	Ergothioneine	Other	Mycelium of *P. linteus*	Antidiabetic	[62]

**Table 2:** The anticancer activity of polysaccharides, hispolon, or others from *P. linteus* in vitro studies.

No.	Polysaccharides, Hispolon, or Others	Model	Dose	Results	References
1	Polysaccharides	Human colorectal carcinoma (HT29) cells	25, 50, 100, 200 μg/mL	Polysaccharides had an inhibitory effect on the proliferation of HT29 cells by blocking the cell cycle in S-phase and downregulation of the expression of cyclin D1, cyclin E, and cyclin-dependent kinases (CDK2), and increased upregulation of the expression of P27kip1 in vitro.	[34]
2	Polysaccharides	HepG2 cells	50, 100, 200 μg/mL	Polysaccharides had an inhibitory effect on the proliferation of HepG2 cells by blocking tumor cells going into the S-phase, upregulating the expression of P27kip1 and cyclin A, and downregulating the expression of calreticulin, cyclin D1, cyclin E, and CDK2 in vitro.	[35]
3	Polysaccharide (PL-N1)	HepG2 cells	50, 100, 200 μg/mL	Polysaccharides had an inhibitory effect on the growth of HepG2 cells.	[36]
4	Polysaccharide (PLPS1 and PLPS-2)	S-180 sarcoma cells	25 μg/mL	Polysaccharides exhibited strong anticancer activity against S-180 sarcoma cells.	[37]
5	Polysaccharides	HepG2 cells	0.5–2.0 mg/mL	These results provided information on significant proteins of hepatocellular carcinoma (HCC).	[38]
6	Polysaccharides	Colon cancer HCT116 and HT29 cells	50 μg/mL	Polysaccharides could reduce the side effects of camptothecin 11 (CPT 11) (10 ng/ml) when they were used as drug combinations.	[39]
7	Ethanol extracts, ethyl acetate extracts, *n*-hexane fractions	HT29 cells	149.9, 69.8, and 77.8 µg/mL	Fractions of ethanol, ethyl acetate, and *n*-hexane inhibited the growth of HT29 cell lines.	[40]
8	Hispolon	NB4 human leukemia cells	10 μg/mL	Hispolon inhibited cell proliferation and promoted cell apoptosis through blocking G/G1 to S transition.	[41]
9	Hispolon	B16-F10 cells	10 μg/mL	Hispolon could induce cell apoptosis by increasing the expression of caspase-3, -8, and -9.	[42]
10	Hispolon	HONE-1 and NPC-039 human nasopharyngeal carcinoma cells	0–100 μM	Hispolon could inhibit cell proliferation and directly induce cell apoptosis by promoting the phosphorylation of JNK1/2, ERK1/2, and p38 MAPK to activate the Csp-3, Csp-8, Csp-9, and Poly ADP-Ribose Polymerase (PARP) expression in a dose- and time-dependent manner.	[43]
11	Hispolon	MCF7 and T47D human breast cancer cells	0–100 μM	Hispolon could induce cell apoptosis through increasing PARP cleavage and decreasing the expression of Bcl-2 and inhibit cell proliferation by reducing the ER-α expression at the level of both mRNA and protein.	[44]
12	Hispolon	Human colon cancer cells	25 µM	Hispolon could induce cell apoptosis.	[45]
13	Hispolon	Human epithelial cancer cells	1–500 mM	Hispolon could inhibit cell proliferation through repressing the transforming growth factor β (TGF-β)-Snail/Twist signaling pathway of epithelial–mesenchymal transition (EMT).	[46]
14	Hispolon	Glioblastoma U87MG cells	25, 50 μM	Hispolon significantly inhibited the tumor cell proliferation and promoted cell apoptosis.	[47]
15	Ethanol extracts	B16F10 melanoma cells	250–500 μg/mL	Ethanol extracts had antiproliferative activity against B16F10 melanoma cells through inducing G0/G1 cell cycle arrest through decreasing cyclin D1 and CDK2 expression and inducing p21.	[48]
16	Extracts	PC-3, DU-145, LNCaP, T24, ACHN, A549, MCF-7, AGS, HepG2, and U-87 cancer cells	0–700 µg/mL	Extracts could induce apoptosis through oxidative stress by stimulating Csp-3 and Csp-9 in varieties of human malignancies, compared with the untreated control.	[49]
17	Aqueous extracts	MDA-MB-231 breast cancer cells	40 mg/mL	Aqueous extracts exhibited an antiproliferative effect with an IC_50_ value of 40 mg/mL in a dose-dependent manner (control: 10 µg/mL 5-flurouracil (5-FU).	[50]
18	Ethanol extracts	G12VKRAS mutant colon cancer cells	100 µg/mL	Ethanolic extracts and cetuximab (10, 30 µg/mL) were combined, and treatment for three days inhibited G12VKRAS mutant colon cancer cells by inducing apoptosis.	[51]
19	3,4-Dihydroxybenzalactone	Human non-small cell lung carcinoma A 549 cells	0, 6.25, 12.5, 25, 50 µM	3,4-Dihydroxybenzalactone inhibited migratory and invasive abilities of cancer cells.	[53]
20	Phellinulin A	Rat hepatic stellate cells	40 µM	Phellinulin A had significant inhibitory and therapeutic effects.	[54]
21	Atractylenolide I	HT29 human colon cancer cells	0–100 µg/mL	Atractylenolide I had good preventive and therapeutic effects.	[40]
22	Hispidin	BxPC-3 pancreatic cancer cells and CSCs	50, 100, 150 μM	Hispidin had therapeutic potential against BxPC-3 pancreatic cancer cells and Cancer stem cells (CSCs) by downregulating the expression of NF-ĸB, in vitro, in a dose-dependent manner.	[55]
23	Phellifuropyranone, meshimakobnol A and meshimakobnol B	Mouse melanoma cells and human lung cancer cells	5.6–31.3 μM, 7.1–22.6 μM and 6.1–15.0 μM	Phellifuropyranone, meshimakobnol A, and meshimakobnol B exhibited antiproliferative activity against mouse melanoma cells and human lung cancer cells in vitro.	[56,57]

**Table 3:** The anticancer activity of polysaccharides, hispolon, or others from *P. linteus* in vivo studies.

No.	Polysaccharides, Hispolon, or Others	Model	Dose	Results	References
1	Polysaccharides	HT29 cells -bearing mouse	100, 200 mg/kg/d	Polysaccharides had an inhibitory effect on tumor growth in a human colorectal carcinoma cell (HT29)-bearing mouse in vivo.	[34]
2	Polysaccharides	HepG2 cells bearing mouse	100, 200 mg/kg	Polysaccharides had an inhibitory effect on tumor growth in a HepG2 cell-bearing mouse in vivo.	[35]
3	Ethanol extracts	C57BL6 mice	300 mg/kg/d	Ethanol extracts reduced tumor weight and increased life span (ILS% = 50.88%) compared with the tumor control group.	[48]
4	Ethanol extracts	Tumor-xenografted mouse	400 mg/kg/d	Ethanol extracts could inhibit the proliferation with a tumor-xenografted mouse model compared with the cetuximab (10, 30 mg/kg/d) control group.	[51]
5	Water extracts	Pancreatic cancer patients	1100 mg 3 times per day	Water extracts could assist the chemotherapy treatment of pancreatic ductal adenocarcinoma, which improved patient survival.	[52]

## Abbreviations

The following abbreviations are used in the manuscript:

*P. linteus*	*Phellinus linteus*
5-Fu	5-Fluorouracil
ABCA1	Adenosine 5′-triphosphate (ATP) binding cassette transporterA1
ABCG1	ATP-binding cassette G1
ABTS	2,2′-azino-bis(3-ethylbenzothiazoline-6-sulfonic acid
ACOX1	Acyl-CoA oxidase 1
AKT	Protein kinase B
ALX	Alloxan
AMG	Aminoguanidine
AP-1	Activator protein-1
ARE	Antioxidant response element
BDE_d_	Bond dissociation enthalpy
CAT	Catalase
CDK2	Cyclin-dependent kinases
CHOP	Kinase-homologous protein
DMN	Dimethylnitrosamine
DOX	Doxorubicin
Cox-2	Cyclooxygenase-2
CPT 11	Camptothecin 11
DBL	3,4-dihydroxybenzalacetone
DPPH	2,2-diphenyl-1-picrylhydrazyl
DSS	Dextran sodium sulfate
EMT	epithelial–mesenchymal transition
EPA	Epalrestat
ERK	Extracellular regulated protein kinase
FAK	Focal adhesion kinase
FRAP	Ferric reducing ability of plasma
Gab2	GRB2-associated-binding protein 2
GPx	Glutathione peroxidase
GSH	Glutathione
GSK3 β	Glycogen synthase kinase-3beta
HCC	Hepatocellular carcinoma
HFD	High-fat high-fructose diet
HO-1	Heme oxygenase-1
HSCs	Hepatic stellate cells
IC_50_	Half maximal inhibitory concentration
IFN-γ	Interferon-gamma
IgE	Immunoglobulin E
IL-1β	Interleukin IL-1β
IL-6	Interleukin IL-6
iNOS	Nitric oxide synthase
KRAS	Kirsten rat sarcoma viral oncogene homolog
LNCaP	Prostate cancer metastasized to lymph node
LPS	Lipopolysaccharides
LPA	Lipoteichoic acid
MAPKs	Mitogen-activated protein kinases
MDA	Malondialdehyde
MITF	Microphthalmia-associated transcription factor
MIC	Minimum inhibition concentration
MMP-9	Matrix metalloproteinase-9
α-MSH	α-melanocyte-stimulating hormone
NO	Nitric oxide
Nrf2	NF-E2-related factor 2
NF-kB	Nuclear factor kappa beta
PA	Palmitate
PARP	Poly ADP-Ribose Polymerase
PC-3	Prostate cancer metastasized to bone
PCET	Proton-coupled electron transfer
PEMs	Peritoneal exudate macrophages
PI3K	Phosphoinositide-3-kinase
PKC	Protein kinase C
PKCδ	Phosphorylation of protein kinase Cδ
PPAR	Peroxisome proliferator-activated receptor
PPI	Protein–protein interaction
PTK	Protein tyrosine kinase
ROS	Reactive oxygen species
SOD	Superoxide dismutase
STZ	Streptozotocin
Syk	Spleen tyrosine kinase
TAA	Thioacetamide
TB	Tumor-bearing
TCM	Traditional Chinese medicine
TEAC	Trolox-equivalent antioxidant capacity
TGF-β	Transforming growth factor β
Th1	T helper 1
Th2	T helper 2
TLR4	Toll-like receptor 4
TNF-α	Tumor necrosis factor
TRAIL	Tumor necrosis factor-related apoptosis-inducing ligand
